# Differentiation and comorbidity of bipolar disorder and attention deficit and hyperactivity disorder in children, adolescents, and adults: A clinical and nosological perspective

**DOI:** 10.3389/fpsyt.2022.949375

**Published:** 2022-08-11

**Authors:** Anna Comparelli, Lorenzo Polidori, Giuseppe Sarli, Andrea Pistollato, Maurizio Pompili

**Affiliations:** ^1^Department of Psychiatry, Sant'Andrea Hospital of Rome, Rome, Italy; ^2^Psychiatry Residency Training Program, Faculty of Medicine and Psychology, Sapienza University of Rome, Rome, Italy; ^3^Department of Neurosciences, Mental Health and Sensory Organs, Faculty of Medicine and Psychology, Suicide Prevention Centre, Sant'Andrea Hospital, Sapienza University of Rome, Rome, Italy

**Keywords:** bipolar disorder, ADHD, comorbidity, nosology and classification of mental disorders, neurodevelopment

## Abstract

Bipolar Disorder (BD) and Attention Deficit and Hyperactivity Disorder (ADHD) are mental disorders with high degree of lifetime comorbidity. Both BD and ADHD are disorders with onset in childhood and early adolescence. Both disorders are often undiagnosed, misdiagnosed, and sometimes overdiagnosed, leading to high rates of morbidity and disability. The psychiatric and behavioral symptoms associated with ADHD and BD have significant overlap. Albeit the existence of a large body of literature, it is far from being clear whether comorbidity can be explained by the confounding overlap of operationally defined criteria or whether it reflects a genuine comorbidity of two biologically distinct disorders. The aim of this paper is to recognize and/or differentiate the pattern of ADHD across the course of BD from a nosological point of view, focusing on specific clinical and neurobiological dimensions. We found that some critical issues may help to fulfill the purpose of our perspective. We suggest that the relationship between ADHD and BD, based on clinical, developmental, and epidemiological commonalities, can be better clarified using four different scenarios.

## Introduction

Bipolar Disorder (BD) and Attention-Deficit/Hyperactivity Disorder (ADHD) are often recognized as mental disorders with a high degree of comorbidity (1–4). Bipolar disorders are chronic diseases characterized by the alternance of episodes of mania or hypomania and depression. Bipolar disorders have an early mean age of onset and are a major cause for disability in young people. Early recognition of the disease leads to more effective treatment and prevents greater disability (5). The definition of bipolar disorder in pre-pubertal children (pediatric bipolar disorder, PBD) is still the object of an ongoing debate. There is a controversy concerning what is the definition of a pre-pubertal bipolar disorder, if there is a continuity of pediatric BD with adult BD, and if symptoms explained by a diagnosis of PBD could be better explained by other diagnoses, such as ADHD (6, 7).

ADHD is defined by the DSM-5 as an early-onset “neurodevelopmental disorder defined by impairing levels of inattention, disorganization and/or hyperactivity-impulsivity” (8). ADHD is a common childhood mental health disorder, even if its prevalence varies widely across different clinical settings and countries, partly due to differences in classifications and methods to diagnose it (9). About 50–65% of children with ADHD will continue to meet diagnostic criteria for ADHD in adulthood (4, 10).

There is an overlap between symptoms of ADHD and BD, and in particular (hypo) manic mood episodes features, such as hyperactivity, distractibility, lack of inhibition, restlessness, racing thoughts, rapid speech, talkativeness, and irritability. The overlap of symptoms and diagnostic criteria could make the distinction between the two disorders difficult, even if ADHD is a disorder with persistent symptoms, whereas symptoms in BD are episodic (4, 11, 12).

Many children are diagnosed with comorbid ADHD and PBD; comorbidity rates are estimated to be around 20% (1, 4), and children with ADHD are more likely to be diagnosed with BD later in life (4). BD with comorbid ADHD presents a more severe course of illness, with earlier onset, a shorter interval between episodes, and a shorter time of euthymia (11).

In this perspective paper, we focused on specific clinical and developmental dimensions in order to recognize and/or differentiate the pattern of ADHD across the course of BD in a nosological perspective. We found that some issues may help to fulfill the purpose of our perspective; we described the results in sections according to the crucial points raised.

## Shared background between BD and ADHD

There is a well-established psychopathological bond between ADHD and BD, even if some studies show controversial results regarding their longitudinal relationship. Rates of comorbidity are very heterogeneous; this is influenced by several aspects. Interestingly, a difference can be detected between continents. For BD, it was suggested that the prevalence may be higher in the Americas compared with Asia and European nations (13, 14); this is not the same for ADHD. Geographic factors only seem to play a marginal role, while all the important differences between countries might be better explained by the adoption of different methodological approaches (9). A high proportion of ADHD patients, about 10%, receive a BD diagnosis throughout their life (2). One out of 13 patients with ADHD have BD, and nearly 1 in 6 patients with BD are diagnosed with ADHD. The prevalence of ADHD in BD patients differs when comparing distinct age groups: 73% in childhood, 43% in adolescence, and 17% in adulthood (3). The lifetime prevalence of ADHD is around 6.5%, while that of BD reaches 1–2% (9, 15, 16). All these data suggest that comorbid BD and ADHD can occur in around 0.12% of the general population, or up to 0.38% if considering smaller studies; this would correspond to nearly 4 million persons affected in US and Europe (16), representing a large burden as a comorbid nosological entity.

There are genetic implications involved in ADHD and BD comorbidity. Relatives of patients with BD had a significantly higher chance of having ADHD, and among relatives of ADHD patients BD occurred more frequently (17); the relative risk was about 2-fold higher for both situations. The existence of a familiarity suggests a genetic vulnerability for both the diseases. In fact, up to 33 loci were found to be involved in both ADHD and BD (18–21).

Shared additional risk factors are related to the prenatal, perinatal, and childhood periods. Maternal substance abuse exposes children to both conditions, and one study showed that this risk factor can be associated with the symptoms of ADHD, but not with a clinical diagnosis (22, 23). Maternal stress exposure during the first trimester was found to correlate with an increased risk of BD (22); the same association emerged with ADHD (24). However, this finding can be also attributed to genetic factors. Mothers suffering from BD or ADHD might experience more maternal stress; genetic background could indirectly impact the offspring (25). Individuals with childhood adversities and trauma were found more likely to develop ADHD and BD (26); regarding the latter, this risk factor may also lead to worse clinical outcomes (27).

## ADHD: Developmental trajectories

The clinical course of ADHD to adulthood is characterized by the development of several psychiatric comorbidities during the transition from childhood/adolescence, impairment of social functioning, and deviant or rule-breaking behaviors. In adolescence, distinct cognitive profiles may emerge both reflecting continuation or potential recovery of ADHD, with or without an emotional dysregulation profile (28). Five different neurobiological developmental models explain the highly variable course of the symptomatology of ADHD during the transition from childhood to adolescence have been supposed (29). These models try to disentangle the reasons that define remission or persistence of symptoms. The first—“convergence”—posits that improvement follows the convergence of atypical neural features toward more typical brain features. The second—“compensation”—views symptomatic improvement as a consequence of the recruitment of new brain systems, compensating the core symptoms of ADHD. The third model—“carried forward”—postulates different adolescent neural trajectories that take origin during childhood. Adolescents showing improvement or remission appear to have more typical neural features, while those showing persistence had more childhood anomalies. The fourth model—“cascading anomalies”—holds that early symptoms of ADHD may worsen neural anomalies and generate neural dysfunction. The last model—“fixed anomalies”—hypothesizes that the presence of childhood ADHD may be seen as a neural imprint that will persist regardless of the clinical course during the transition toward adolescence.

Several clinically informed factors such as gender, pre-mature birth, maternal education, school readiness, peer and conduct problems, cognitive and temperamental factors, early comorbidities, social context and medication could differentiate individuals following different ADHD symptom trajectories (29, 30). Genetic factors are major drivers for ADHD symptoms persistence during adolescence (31); conduct problems and comorbidities seems to play a major role for the future development of Bipolar Disorder (32).

## Clinical trajectories from childhood ADHD to adult ADHD: Syndromatic and symptomatic ADHD

A large body of literature (28–30) suggests that ADHD tends to persist from childhood to adulthood in many cases. Some questions have been raised about the need for a proper definition of “persistence.” According to Faraone et al. (29), syndromatic persistence is the permanence of a full diagnostic status, referring to individuals continuing to meet ADHD diagnostic criteria, even after the adulthood transition. On the other hand, symptomatic persistence is the presence of a partial diagnostic status along with impairment, referring to individuals who fail to meet full blown diagnostic criteria for ADHD, but who continue to have impairing symptoms.

A 10-year follow-up study by Biederman et al. (28) demonstrated that the majority of ADHD individuals continue to experience symptoms and functional impairment when moving into adulthood. Persistence at follow-up was associated with psychiatric comorbidity, functional impairments, familiarity, comorbid Conduct Disorder (CD), Major Depressive Disorder (MDD), treatment for ADHD and Oppositional-Defiant Disorder (33, 34).

A recent global systematic review and meta-analysis (30) estimated the prevalence of persistent adult ADHD (considering a childhood onset) and symptomatic adult ADHD (not considering childhood onset). Both measures showed a decrease with advancing age. Prevalence of persistent adult ADHD was 2.58%; prevalence of symptomatic adult ADHD was 6.76% globally.

## Bipolar disorder developmental trajectory: “Homotypic trajectory” and “heterotypic trajectory”

Many studies, including systematic reviews, meta-analyses, prospective and retrospective studies, have evaluated the developmental trajectory ADHD-BD (2, 11, 32, 35–38).

A recent systematic meta-analytic review (2) estimated that about 10–12% of individuals with ADHD will be later diagnosed with BD; this transition mostly occurs during development. According to another follow-up study including a total of 17,285 subjects with ADHD conducted in Taiwan (35) the progression rate from ADHD to BD was 5.12%. Interestingly, among all participants, 62.16% progressed within the first 3 years, following the ADHD onset. Geographical features and sample sizes could explain progression rates differences. From a retrospective view Nierenberg et al. (11) estimated a 9.5% overall lifetime prevalence of comorbid ADHD in adults with BD.

Predictors of BD, considering the psychopathological characteristics that forewent the disorder, could be evaluated following the model proposed by Faedda et al. (32). More specifically, this involved a “homotypic trajectory” moving from affective psychopathology toward BDI/II, and an “heterotypic trajectory” moving from non-affective psychopathology. Evidence from the aforementioned paper showed a heterotypic developmental trajectory from prodromal sub-syndromal and syndromal Disruptive Behavior Disorders (ADHD, Conduct Disorder) and Anxiety Disorders (early-onset panic attacks, separation anxiety, and social phobia) toward BD. ADHD or anxiety increased the risk of developing BD in adulthood by 10-fold, while the combination of ADHD and anxiety increased the risk by 30-fold (38). This suggests that early manifestations of both externalizing and internalizing psychopathology could be related with the risk of future BD, considering internalizing psychopathology as an expression of conditions associated with negative emotions and externalizing psychopathology as an expression of conditions characterized by disinhibition (39). This dysregulation profile is characterized by a combination of externalizing (inattention and hyperactivity) and internalizing (anxiety) psychopathology that may indicate youth with propensity to BD. Dysregulation profile is currently conceptualized as a broad syndrome of difficulties in regulating affect, behavior, and cognition (40, 41). According to Brancati et al. (2), moving from this pattern of prodromal features emotional dysregulation can be considered as a trans-nosographic psychopathological dimension that facilitates the progression from ADHD to BD.

Several conditions and psychiatric comorbidities elevate the risk of progression from ADHD to BD. Among these are family history of BD, older age, Major Depressive Disorder (MDD), Anxiety Disorder, Autism Spectrum Disorder (ASD), Intelligence Disability (ID), Disruptive Behavior Disorder (DBD), Oppositional-Defiant Disorder (ODD), Conduct Disorder (CD), criminal behavior, Alcohol Use Disorder (AUD), and Cluster A or B Personality Disorder (32, 35, 36, 38).

## Comorbidity between BD and ADHD

There are significant clinical differences between patients diagnosed with both ADHD and BD and patients with BD without comorbid ADHD. Compared with the “pure” BD counterpart, patients with BD and ADHD have shorter period of wellness and stability (37), are younger when experiencing their first psychiatric symptoms (2, 11), show earlier treatment implementation (2) and are less successful at school and more unemployed (42). Talking about behavioral and properly ADHD features, patients with comorbid ADHD-BD exhibit a younger age of ADHD onset (2, 11), more externalizing problems (2, 43), a higher severity of hyperactive/impulsive and inattentive symptoms (2), more reactivity and verbal aggressiveness (44) and a history of violence, legal troubles, and rule-breaking behaviors (2, 37). Moreover, referring only to affective episodes, individuals with comorbid ADHD-BD experience significantly more depressive, mixed, hypo-manic, and total number of mood episodes (2, 37, 45), shorter euthymic intervals (2), earlier age of onset of mood disorder, more severe and chronic mood disorder, poorer response to mood stabilizer, and are more irritable (2, 37, 45, 46); the psychotic features are, however, less likely compared to “pure” bipolar patients (37). At last, for these patients more suicide attempts, more additional psychopathology (Anxiety, Disruptive Behavior Disorders, Substance Use Disorder and Alcohol Abuse) and more psychiatric hospitalizations are documented (2, 3, 11, 47).

A recent systematic review and meta-analysis (16) reported that there is no difference in comorbidity between ADHD and BD type I or BD type II. Nevertheless, differential diagnosis between ADHD and BD type II appears to be challenging due to the presence of sub-syndromal phenomena and mood states such as hypomania, rapid cycling, mixed episodes, and high-frequency mood swings, which are particularly common in BD type II. The trait-like nature of ADHD must be considered when comparing with state-like features of BD. A positive history of childhood ADHD can impact both the phenotype and the onset of depressive disorder, increasing the risk of bipolar spectrum features. Screening for lifetime ADHD in depressed individuals with treatment refractoriness and mixed features should be mandatory; on the other hand, young adults with ADHD along with a familiar load for mood disorder or with anxiety disorders should be candidates for depressive disorder prevention (36).

## Discussion

Herein, we aimed to focus on specific clinical, developmental and neurobiological dimensions to recognize and/or differentiate the pattern of ADHD across the course of BD from a nosological perspective.

We are inclined to propose four scenarios that aim to clarify possible different relationships in clinical and research settings:

1. Overestimated comorbidity of ADHD or BD due to overlap of symptoms (especially in childhood and youth);
2. ADHD can be a prodrome of BD;
3. Comorbidity of syndromatic ADHD with strictly defined BD;
4. ADHD-BD is a full entity.

Regarding the first point, some authors (48, 49) have highlighted the possibility of a diagnostic artifact rather than a genuine finding when considering BD and ADHD in comorbidity. The adoption of a categorical approach instead of a dimensional one, over-splitting of symptoms (artificial subdivision of syndromes), and the overlapping symptoms such as impulsivity, irritable mood, and poor concentration might contribute to the development of a “false” comorbidity. In childhood and youth, differential diagnosis is clinically the most difficult due to the large overlap of the symptomatic and family patterns of both disorders; currently, there is substantial consensus that episodes are one of the hallmarks of BD and that phenotypes characterized by chronic irritability and lack of episodicity, are not consistent with a diagnosis of BD (50). In adulthood, a correct differential diagnosis between BD and ADHD, is pivotal, because irritability, inattention and hyperactivity improve with specific medications, and patients often do not need a mood stabilizer (that might be given with an overestimated comorbidity of BD) (51). A wrong BD diagnosis in adults with ADHD can lead to mood stabilizers that do not usually improve attention and memory, whilst the stimulant therapy can improve overall symptomatology, including irritability and hyperactivity (52).

Considering the second scenario, misleading comorbidity may be also related to developmental sequencing, which is an example of “heterotypic continuity” in which the same developmental process has different phenotypes at different stages of life. In this case, ADHD may represent a BD precursor in a heterotypic trajectory. Unfortunately, we are not currently able to accurately predict which patients with ADHD are prone to subsequent development of BD. In fact, on one hand, a specifically increased risk of BD can be confirmed in ADHD patients compared to healthy controls, which is higher than what expected based on the general predisposing effect on other kinds of psychopathology. On the other hand, BD following ADHD may be a specific, pediatric-onset, neurodevelopmentally-based, form of BD, different from adult-onset or older-age (neurodegenerative)-onset BD, despite phenotypical similarities. Early neurodevelopmental disorders, such as co-occurring ADHD and emotional dysregulation, may be considered a precursor in the pathway to neurodevelopmental, early-onset, bipolarity (2).

As regard to the third scenario, although the higher known rates of ADHD and BD comorbidity is in childhood (3), we suggest that further studies are needed to establish the timing of emergence of different forms of BD in ADHD patients and *vice-versa*. Lower but still rather high comorbidity rates have been reported at later ages. Therefore, in adult patients with a recent diagnosis of BD, but with an unknown history of ADHD, an accurate assessment for a previous or current diagnosis of ADHD appears mandatory (3); if a history of childhood or adolescent ADHD emerges, clinicians should carefully ascertain whether the ADHD persists in a symptomatic or syndromatic form; only in the latter should a diagnosis of comorbidity be made (32).

The hypothesis of a full entity ADHD-BD, different from both ADHD and BD, is supported by several familial (53, 54) and genetic (55) studies. These findings suggest a mixed BD-ADHD disorder, with its own evolution, continuous rather than cyclic (56), earlier onset, male predominance, more frequent episodes with mixed states, irritability as a main feature, more severe manic symptoms, increased psychosocial problems, and necessitating a sequential treatment. Interestingly, this hypothesis is also in line with recent dimensional approaches to nosology, such as the Hierarchical Taxonomy of Psychopathology (HiTOP), which aims to identify psychopathology constructs based on patterns of co-variation among signs and symptoms (57), in contrast to the DSM/ICD nosography. In a dimensional perspective, in fact, ADHD and bipolar disorder may fall on a genetic continuum of severity, with patients with ADHD but not bipolar disorder being at the mild end, patients with bipolar disorder but not ADHD having greater severity and patients with both ADHD and bipolar disorder having the greatest severity (58).

Apart from theoretical and nosological issues, therapeutic implications may arise from perspective paper such as the present one.

Treatment of concurrent ADHD and BD, regardless of the phase of illness, remains an unresolved challenge; in a developmental perspective view, treatment may require a staged approach. By staging the introduction of treatments, one can reduce the risk of overmedicating patients and better assess the effect of each individual treatment (59). On the other hand, in case of real comorbidity, a hierarchical approach to treatment should be followed. At present, data on treatment response of comorbid ADHD-BD are very limited. In clinical practice, most adult patients presenting with symptoms of both ADHD and BD tend to be treated only for the mood disorder (60). The use of BD medications in ADHD-BD patients may be problematic, with high rates of non-response and residual functional impairment. Patients should be treated hierarchically: BD should be treated first, while ADHD should be treated combining ADHD medications and mood stabilizers after mood stabilization (61). A proper mood stabilizing therapy can reduce the chance of positive mood episodes that might arise if we only use ADHD specific medications and that is why we actually follow a hierarchical approach.

Our perspective is focused on the clinical and developmental aspects of ADHD and BD; genetic, neurobiological, neurocognitive and therapeutic aspects were not part of the aims of this perspective paper. We hope that better clinical and developmental characterization is coming and that parallel efforts through nosology can be useful.

## Data availability statement

The original contributions presented in the study are included in the article/supplementary material, further inquiries can be directed to the corresponding author.

## Author contributions

AC: conception, original draft, and revision. LP, GS, and AP: drafting and collecting data. MP: final revision. All authors contributed to the article and approved the submitted version.

## Conflict of interest

The authors declare that the research was conducted in the absence of any commercial or financial relationships that could be construed as a potential conflict of interest.

## Publisher's note

All claims expressed in this article are solely those of the authors and do not necessarily represent those of their affiliated organizations, or those of the publisher, the editors and the reviewers. Any product that may be evaluated in this article, or claim that may be made by its manufacturer, is not guaranteed or endorsed by the publisher.
